# Ell3 stabilizes p53 following CDDP treatment via its effects on ubiquitin-dependent and -independent proteasomal degradation pathways in breast cancer cells

**DOI:** 10.18632/oncotarget.5972

**Published:** 2015-10-19

**Authors:** Hee-Jin Ahn, Kwang-Soo Kim, Kyung-Won Shin, Kee-Hwan Lim, Jin-Ock Kim, Je-Yong Lee, Jiewan Kim, Ji-Hoon Park, Kyung-Min Yang, Kwang-Hyun Baek, Jeong-Jae Ko, Kyung-Soon Park

**Affiliations:** ^1^ Department of Biomedical Science, College of Life Science and CHA Stem Cell Institute, CHA University, Seoul, Korea; ^2^ Graduate School of Biomedical Science, CHA University, Seoul, Korea; ^3^ Department of Biomedical Science, College of Life Science and CHA Cancer Institute, CHA University, Seoul, Korea

**Keywords:** Ell3, p53, cis-diamminedichloroplatinum(II) (CDDP), NAD(P)H quinone oxidoreductase 1 (NQO1), interleukin-20 (IL20)

## Abstract

The tumor suppressor protein p53 is unstable in quiescent cells and undergoes proteosomal degradation. Under conditions of cellular stress, p53 is rapidly stabilized by post-translational modification, thereby escaping degradation and translocating to the nucleus where it activates genes related to cell cycle arrest or apoptosis. Here, we report that the transcription elongation factor Ell3 sensitizes luminal type-cancer cell line, MCF7, which have wild-type p53, to the chemotherapeutic agent cis-diamminedichloroplatinum(II) (CDDP) by stabilizing p53. Overexpression of *Ell3* in MCF7 cells suppressed the MDM2-mediated ubiquitin-dependent degradation pathway. In addition, Ell3 promoted binding of p53 to NADH quinone oxidoreductase 1, which is linked to the ubiquitin-independent degradation of p53. We found that Ell3 activates interleukin-20 (IL20) expression, which is linked to the ERK1/2 signaling pathway. Chemical inhibition of ERK1/2 signaling or molecular suppression of IL20 revealed that the ERK1/2 signaling pathway and IL20 are the main causes of p53 stabilization in Ell3-overexpressing MCF7 cells. These findings suggest that the ERK1/2 pathway can be targeted in the rational development of therapies to induce chemosensitization of breast cancer cells.

## INTRODUCTION

*ELL3* belongs to the eleven-nineteen lysine-rich leukemia (*ELL*) family of RNA polymerase II transcription elongation factors and is enriched in testis. In mice, Ell3 primes gene activation during embryonic stem cell (ESC) specification by marking the enhancers of developmentally regulated genes, thereby establishing PolII localization [1]. Recently, our laboratory reported that Ell3 enhances the differentiation of mouse ESCs by protecting differentiating cells from apoptosis through the promotion of p53 degradation [2].

p53 plays a central role in oncogenesis by regulating the cell cycle, DNA repair, and apoptosis. Most types of human cancers have a mutated p53 pathway, highlighting its role in tumorigenesis [3]. p53 is maintained at low levels in unstressed cells through continuous proteasomal degradation by both ubiquitin-dependent and ubiquitin-independent processes. While ubiquitin-dependent degradation of p53 is an active process, ubiquitin-independent degradation is a passive process. Unstructured p53 is inherently unstable and is degraded through the 20S proteasome by default unless inhibited by NAD(P)H quinone oxidoreductase 1 (NQO1). NQO1 is a flavin-containing quinone reductase catalyzing the reduction of various quinones using either NADH or NADPH as a reducing cofactor [4]. p53 is stabilized by binding to NQO1 in an NADH-dependent process [5, 6]. In the presence of dicumarol, curcumin, and several other inhibitors of NQO1 activity, p53 becomes highly unstable and prone to proteasomal degradation.

Many chemotherapeutic regimens employed for the treatment of breast cancer include platinum-based drugs, in particular cis-diamminedichloroplatinum(II) (CDDP, best known as cisplatin). The anticancer activity of CDDP is initiated through its binding to DNA, causing crosslinking and subsequently generating DNA lesions. The DNA damage interferes with replication and activates the DNA damage response and apoptotic machinery. Despite initial efficacy, CDDP treatment often results in the development of chemoresistance, leading to therapeutic failure. Intense research has been conducted to understand this chemoresistance mechanism with aim of developing chemosensitization strategies. It is currently known that CDDP induces MAP kinase pathways in ovarian carcinoma cell lines [7]. Inhibition of CDDP-induced ERK1/2 activity by the chemical inhibitor PD98059 decreases the half-life of p53, which causes the cells to become chemoresistant [8].

Cytokine interleukin 20 (IL20) is associated with inflammatory diseases such as rheumatoid arthritis, contact hypersensitivity, and atherosclerosis [9]. IL20 induces cellular responses through a type I receptor composed of IL20R/IL20R2 chains and a type II receptor consisting of an IL22R1/IL20R2 heterodimer [10, 11]. IL20 transmits its signal through signal transducer and activator of transcription 3 (STAT3) in keratinocytes [12]. IL20 treatment activates MAPK signaling proteins, such as ERK1/2, p38 MAPK, and JNK, in human umbilical vein endothelial cells. IL20 stimulation of GBM8901 glioblastoma cell cultures activates the JAK2/STAT3 and ERK1/2 pathways [13, 14]. IL20 promotes migration and invasion of bladder cancer cells through ERK-mediated MMP-9 protein expression [15], and increases the cell proliferation and colony formation of oral cancer cells by activating STAT3 and ERK signaling [16]. Although IL20 is associated with tumor progression through its regulation of migration and invasion, little is known about its role in the response of tumor cells to drugs.

In this study, we found that Ell3 sensitized MCF7 cells to CDDP by stabilizing p53 protein. This stabilization was achieved by suppression of the MDM2-mediated ubiquitination pathway. Ell3 also increased p53 binding to NQO1, allowing p53 to avoid the default passive degradation pathway. Furthermore, we found that Ell3 increased IL20 expression, leading to the activation of the ERK1/2 signaling pathway. Ell3 up-regulation of IL20 and ERK signaling is the primary cause of p53 stabilization in response to CDDP treatment.

## RESULTS

### Ell3 sensitizes MCF7 cells to DNA-damaging chemotherapeutics through p53

**Figure 1:**
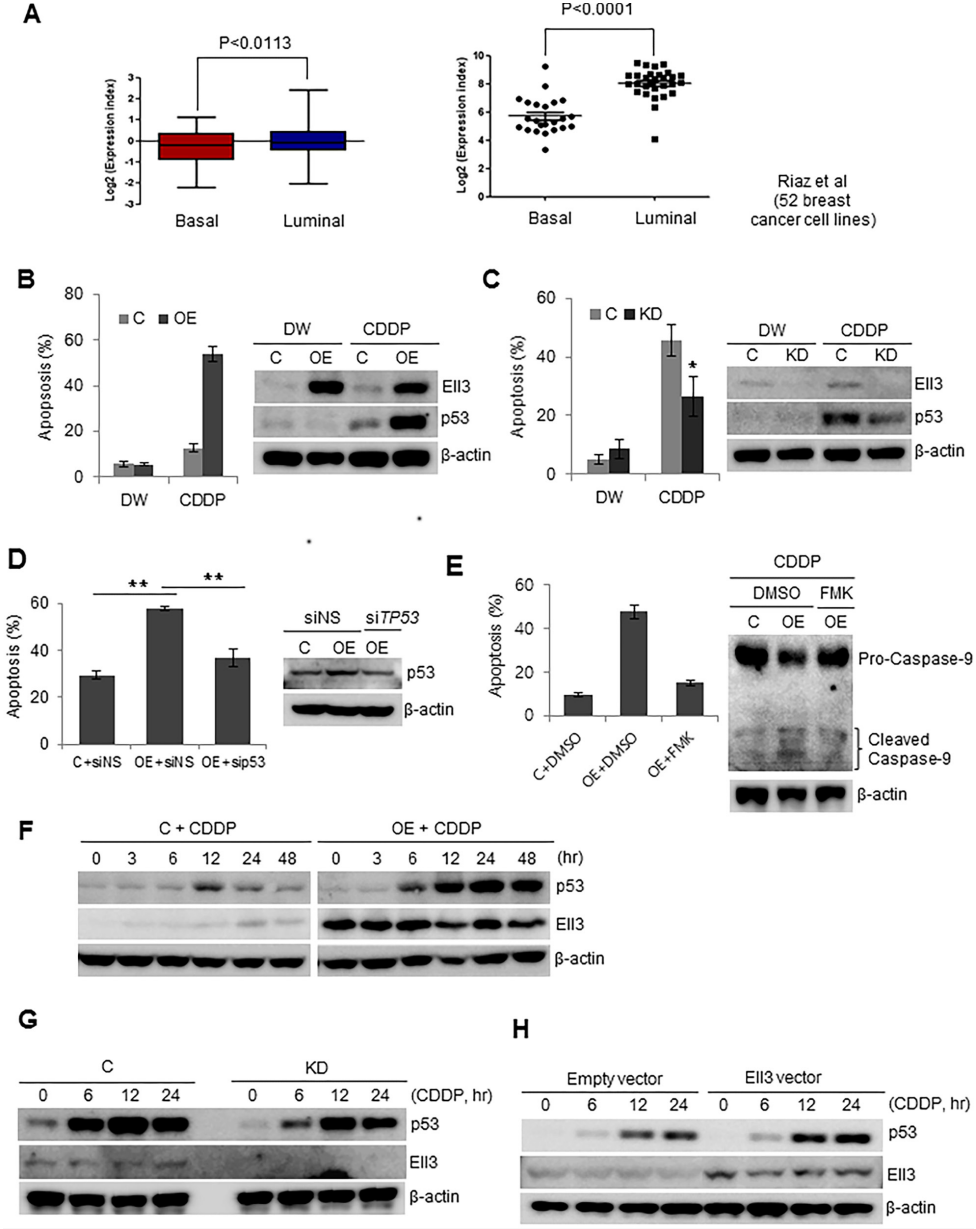

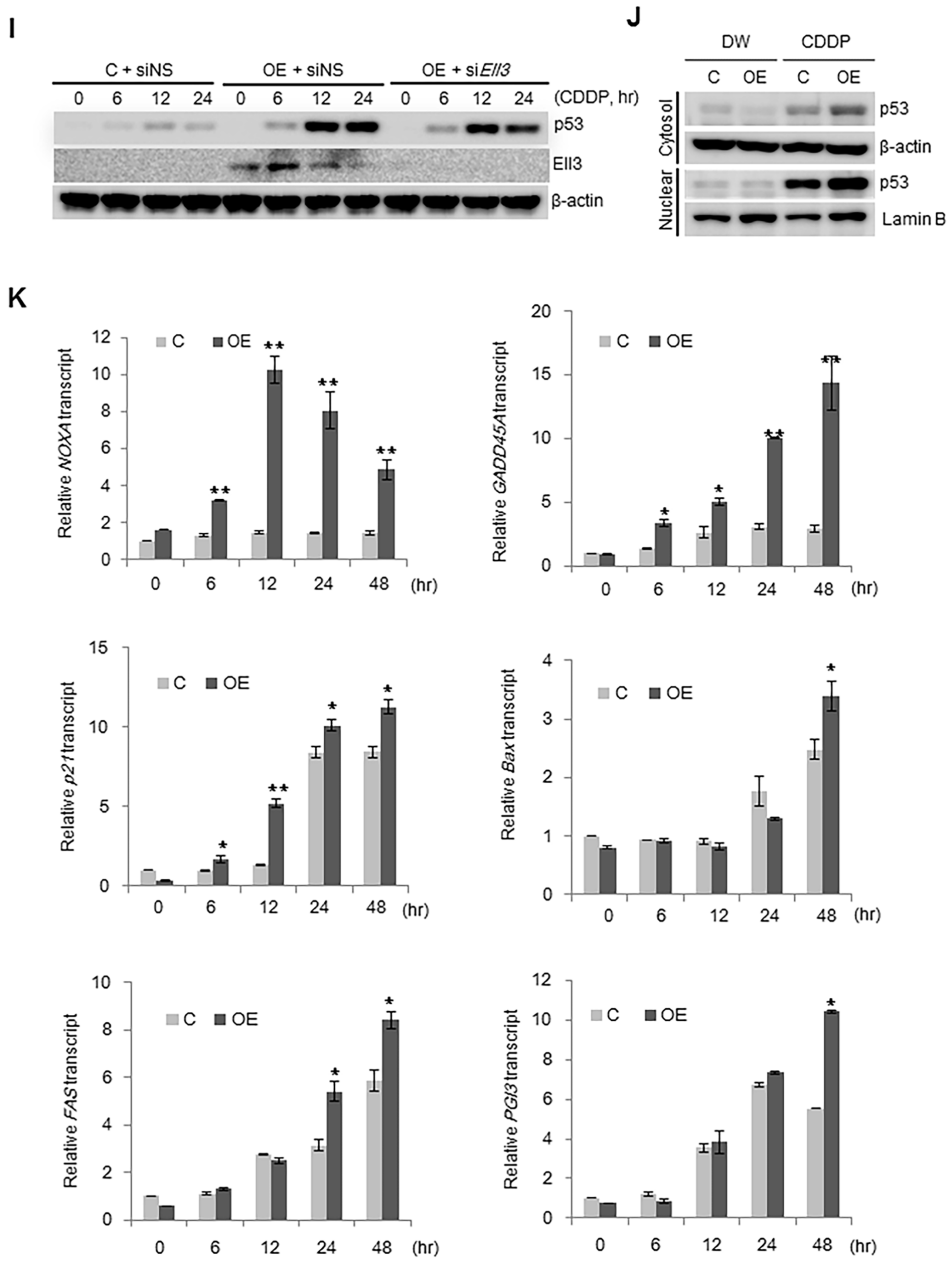
Ell3 sensitizes MCF7 cells to CDDP in a p53-dependent manner **A.** The expression of *Ell3* in resected breast tumors (154 with luminal type and 55 with basal type, left panel) and human breast cancer cell lines (29 luminal and 23 basal, right panel) was analyzed using public microarray datasets. **B.** Apoptosis assayed by flow cytometry (left) and western blotting (right) in Ell3-overexpressing (Ell3-OE) and control MCF7 cells exposed to CDDP (8 μg/ml) or distilled water (DW) for 24 h. **C.** Western blot (right) and apoptosis assay (left) in Ell3-knockdown (Ell3-KD) and control MCF7 cells exposed to CDDP (16 μg/ml) or DW for 24 h. **D.** Apoptosis assay in si*TP53*-transfected and CDDP-treated Ell3-OE. Cells were transfected with non-specific siRNA (siNS) or siRNA targeting *TP53* (si*TP53*) for 24 h. After transfection, cells were exposed to CDDP for 24 h and the annexin V-positive apoptotic fractions and p53 protein levels were compared. **E.** Apoptosis assay (left) and western blot (right) in CDDP-treated Ell3-OE that were pre-exposed to a caspase inhibitor (z-VAD-FAM, 10 μM) for 8 h and CDDP for 24 h. **F.** Western blot of p53 and Ell3 levels at the indicated times during CDDP treatment. To detect p53 protein in Ell3-OE, 20 μg of total protein was analyzed. **G.** Western blot of p53 levels at the indicated times after CDDP treatment in Ell3-KD and control cells. To detect p53 protein in control cells, 50 μg of total protein was analyzed. **H.** The effect of transiently expressed Ell3 on p53 accumulation in MCF7 cells after CDDP treatment. An Ell3 expression plasmid or a control plasmid was transfected into MCF7 cells for 24 h. Transfected cells were treated with CDDP for 24 h and assessed for p53 expression by western blotting. **I.** The effect of siRNA targeting *Ell3* (si*Ell3*) on p53 accumulation during CDDP treatment. Ell3-OE were transfected with non-specific siRNA (siNS) or si*Ell3* for 24 h. Cells were exposed to CDDP at indicated times and then the p53 levels were analyzed by western blotting. **J.** Control cells or Ell3-OE exposed to CDDP were fractionated into cytosolic and nuclear fractions and subjected to western blotting. **K.** *NOXA*, *GADD45A*, and p21 transcripts were quantitatively analyzed by real-time PCR in control and Ell3-OE cells at the indicated times after CDDP treatment. Error bars represent the SE from three independent experiments, each performed with triplicate samples. *P < 0.05, **P < 0.01, Student's *t*-test.

In a previous study, we reported that Ell3 in breast cancer cell lines induces resistance to 5-fluorouracil via a MEK/ERK-dependent signaling pathway [17]. To extend our understanding of the function of Ell3 in breast cancer, we used public microarray datasets to analyze the expression level of the *Ell3* gene in 209 resected breast tumors (GSE2034 [18]) and in 52 human breast cancer cell lines (GSE41313). In breast tumors, a significantly higher level of *Ell3* expression was observed in luminal than in basal tumor types (*P* < 0.0113, Figure 1A, left panel). A similar expression pattern was observed in breast cancer cell lines (*P* < 0.0001, Figure 1a, right panel). To elucidate the meaning of *Ell3* expression in breast cancer cells, we engineered MCF7 cells to overexpress Ell3 and examined the response of these cells to CDDP. CDDP treatment of Ell3-overexpressing MCF7 cells (Ell3-OE) resulted in a hypersensitive response that induced apoptosis and p53 accumulation (Figure 1B). In contrast, in MDA-MB-231 and Hs578T cells, which have mutated forms of p53, both control and overexpressing cells showed an apoptotic response and p53 accumulation when treated with CDDP (Supplementary Figure S1). To determine if the response of Ell3-OE to CDDP was induced by Ell3, we examined the response of stable *Ell3*-knockdown MCF7 cells (Ell3-KD) to CDDP treatment. Contrary to Ell3-OE cells, the extent of apoptosis in Ell3-KD cells was less than that in control cells and was accompanied by lower p53 accumulation (Figure 1C). Furthermore, introduction of p53 siRNA (sip53) decreased CDDP-mediated apoptosis in Ell-OE cells to a level similar to that of control cells, indicating that the apoptosis requires a p53-dependent pathway (Figure 1D). The effect of the expression level of *Ell3* on the apoptotic response of MCF7 cells to CDDP was confirmed by the MTT assay. Consistent with the results of the flow cytometric analysis of apoptotic cells, the MTT assay revealed that Ell3-OE cells are sensitive to CDDP, whereas Ell3-KD cells are resistant to CDDP, compared with control cells (Supplementary Figure S2). Pretreatment with a general caspase inhibitor (z-VAD-FMK) significantly reduced apoptosis from 43.72% ± 1.23% to 18.45% ± 0.63% (*P* < 0.01), indicating that Ell3 induces CDDP-mediated apoptosis in MCF7 cells through caspase activation (Figure 1E). As shown in Figure 1F, p53 gradually accumulated in Ell3-OE in a time-dependent manner during CDDP treatment. However, the p53 level transiently increased in control cells 12 h after CDDP treatment and then returned to basal levels. In Ell3-KD cells, consistent with the apoptotic phenotype, the level of p53 accumulation was lower than that in control cells after CDDP treatment (Figure 1G). Overexpression of *Ell3* in MCF7 cells also induced p53 accumulation after CDDP treatment (Figure 1H, Supplementary Figure S3A). In addition, introduction of siRNA targeting *Ell3* in Ell3-OE cells resulted in lower p53 accumulation at 24 h (Figure 1I, Supplementary Figure S3B). These results indicate that p53 accumulation in MCF7 cells following CDDP exposure is induced by Ell3 activity. Subcellular fractionation analysis showed that p53 translocated to the nucleus following CDDP treatment in Ell3-OE cells (Figure 1J). Consistent with p53 accumulation in Ell3-OE cells as early as 6 h after CDDP treatment, the expression of p53 target genes including *NOXA*, *GADD45A*, and *cyclin-dependent kinase inhibitor 1A* (p21) increased 6 h after CDDP treatment, indicating that accumulated p53 was functionally active and able to induce the expression of target genes (Figure 1K).

### Ell3 increases p53 protein stability

**Figure 2:**
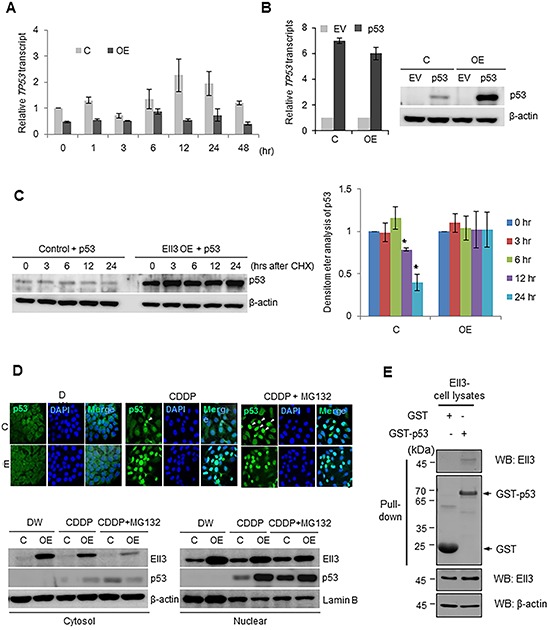
Ell3 enhances p53 protein stability after CDDP treatment **A.** p53 transcripts were quantified by real-time PCR in control and Ell3-overexpressing (Ell3-OE) MCF7 cells at the indicated times during CDDP treatment. **B.** A p53 expression plasmid or empty vector (EV) was transfected into control and Ell3-OE cells for 24 h after which. p53 mRNA and protein levels were analyzed. **C.** A p53 expression plasmid was transfected into control or Ell3-OE cells for 24 h and then cells were treated with 5 μM cyclohexamide (CHX); p53 protein levels were analyzed by western blotting at the indicated times after treatment (left) and quantified by densitometry (right). **D.** Control or/and Ell3-OE cells were exposed to CDDP for 18 h, treated with 5 μM MG132 for 6 h, and then subjected to immunocytochemical staining of p53 (upper) or fractionation into cytosolic and nuclear fractions. **E.** Western blots of a GST pull-down assay. Lysates of HEK 293 cells transfected with Ell3 were reacted with GST or GST–p53 beads. Error bars represent the SE from three independent experiments, each performed with triplicate samples. *P < 0.05, **P < 0.01, Student's *t*-test.

The total amount of p53 protein is strictly regulated both at the transcriptional and post-translational levels. To determine whether the accumulation of p53 in Ell3-OE cells following CDDP treatment was induced by transcriptional activation of *TP53*, we measured *TP53* transcript levels. As shown in Figure 2A, *TP53* transcript levels in Ell3-OE cells were lower than those in control cells and did not significantly change after CDDP treatment. This result suggests that p53 accumulation was caused by a change in protein turnover. Therefore, we transiently overexpressed *TP53* and analyzed its RNA and protein levels in Ell3-OE and control cells. As expected, p53 protein accumulated to a higher level in Ell3-OE cells than in control cells, whereas *TP53* transcript expression was similar (Figure 2B). When p53 protein biosynthesis from the *TP53* expression plasmid was blocked with cyclohexamide, the amount of p53 in Ell3-OE was stably maintained for 24 h whereas the p53 level in control cells gradually decreased (Figure 2C). Figure 2D shows that a large amount of p53 translocated to the nucleus of Ell3-OE cells following CDDP treatment, whereas, in control cells, a comparable amount of p53 accumulated in the nucleus only when MG132 was added to block the proteosomal degradation pathway. These results confirm that Ell3 enhanced the stability of p53 protein by blocking proteosomal degradation. A GST pull-down assay showed that p53 directly binds to Ell3, suggesting that the physical binding of p53 to Ell3 might protect p53 from proteosomal degradation (Figure 2E). Co-immunoprecipitation assays using extracts from deletion mutants of Ell3 and p53 suggest that the whole domain of Ell3 binds to p53 (Supplementary Figure S4).

### Ubiquitin-dependent degradation of p53 is suppressed in Ell3-OE cells

**Figure 3:**
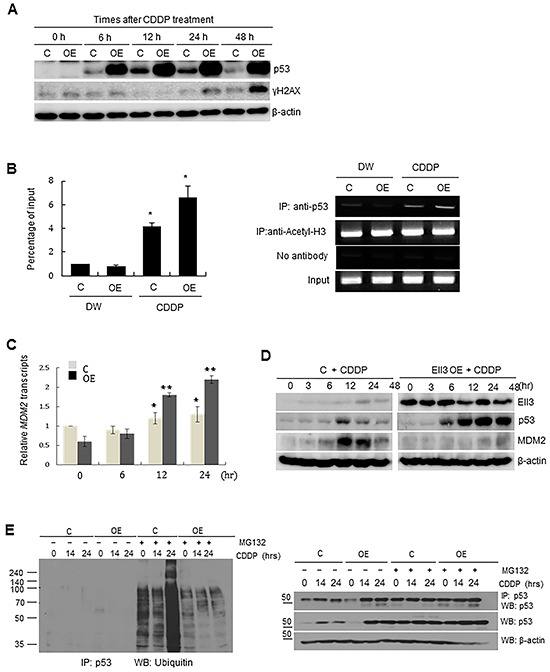
Ubiquitinating enzymes of p53 are suppressed in Ell3-OE cells **A.** The amount of p53 and γH2AX protein in control and Ell3-overexpressing (Ell3-OE) MCF7 cells was analyzed by western blotting at the indicated times after CDDP treatment. **B.** CDDP treatment caused recruitment of p53 to the promoter of *MDM2*: Ell3-OE and control cells were treated with CDDP or distilled water (DW) for 24 h and then the binding of p53 to the *MDM2* promoter was analyzed by chromatin immunoprecipitation (ChIP)-qPCR. Right panel, representative gel images of ChIP experiments using anti-p53 or anti-acetyl-H3 antibody; left panel, Quantitative analysis of percentage of *MDM2*-promoter bound p53. Data are expressed in percentage changes relative to the amount of input DNA. Anti-acetyl-H3 antibody was used as positive control. **C.** *MDM2* transcripts were quantified by performing real-time PCR in control or Ell3-OE cells at the indicated times after CDDP treatment. **D.** The expression of p53 and MDM2 in control and Ell3-OE cells was analyzed by western blotting at the indicated times after CDDP treatment. **E.** Ubiquitination of p53 in control and Ell3-OE cells at the indicated times after CDDP treatment, in the absence or presence of MG132. Total protein from each sample was immunoprecipitated with anti-p53 antibodies and ubiquitinated p53 was detected by western blotting using anti-ubiquitin antibodies (left panel). The amount of immunoprecipitated p53 was analyzed by western blotting with anti-p53 antibodies to confirm that the difference in ubiquitination was not caused by an unequal loading of proteins. Error bars represent the SE from three independent experiments, each performed with triplicate samples. *P < 0.05, **P < 0.01, Student's *t*-test.

We next addressed whether p53 accumulation in Ell3-OE cells was caused by rapid activation of the DNA damage response pathway after CDDP treatment. CDDP-mediated formation of mono-adducts and intra- and inter-strand crosslinks in DNA inhibits DNA replication by causing replication fork collapse [19]. Replication fork collapse leads to single-stranded DNA breaks and activates the DNA damage response, resulting in the activation and accumulation of p53. Unexpectedly, p53 began to accumulate at 6 h after CDDP treatment whereas γH2AX, an indirect marker of DNA strand breaks and replication arrest [20], appeared 48 h after CDDP treatment in Ell3-OE (Figure 3A). This result suggests that p53 stabilized and accumulated before DNA was damaged by CDDP in Ell3-OE cells. Since p53 and MDM2 formed a complex in the presence of Ell3 (Supplementary Figure S4), we next examined whether MDM2-induced p53 ubiquitination is altered in Ell3-OE cells.

Phosphorylation of p53 at serine 15, which stabilizes p53 by impairing its binding to MDM2, increased in parallel with the increase in the total amount p53 in Ell3-OE cells following CDDP treatment (Supplementary Figure S5). p53 and MDM2 form an autoregulatory loop, in which MDM2 degrades p53 through a ubiquitin-dependent pathway and p53 functions as a transcriptional activator of *MDM2*. Indeed, chromatin immunoprecipitation (ChIP) analysis showed that p53 bound to the promoter of MDM2 in the presence of CDDP in both Ell3-OE and control cells (Figure 3B). Consistent with the results of ChIP analysis, the level of *MDM2* transcripts increased following CDDP treatment in both Ell3-OE and control cells (Figure 3C). Despite the activation of *MDM2* transcription by CDDP, MDM2 protein did not accumulate in Ell3-OE cells (Figure 3D). Consistent with the deficiency of MDM2 protein, ubiquitination of p53 in Ell3-OE was weaker than that in control cells, and MDM2 ubiquitination of p53 did not increase after CDDP treatment in Ell3-OE cells (Figure 3E). These results suggest that the feedback pathway regulating p53 degradation might be impaired in Ell3-OE regardless of the activation of *MDM2* transcription. Taken together, we conclude that an impaired p53 ubiquitination system, in part, contributes to p53 accumulation in Ell3-OE cells following CDDP treatment.

### p53 is stabilized by its binding to NQO1 in Ell3-OE cells

**Figure 4:**
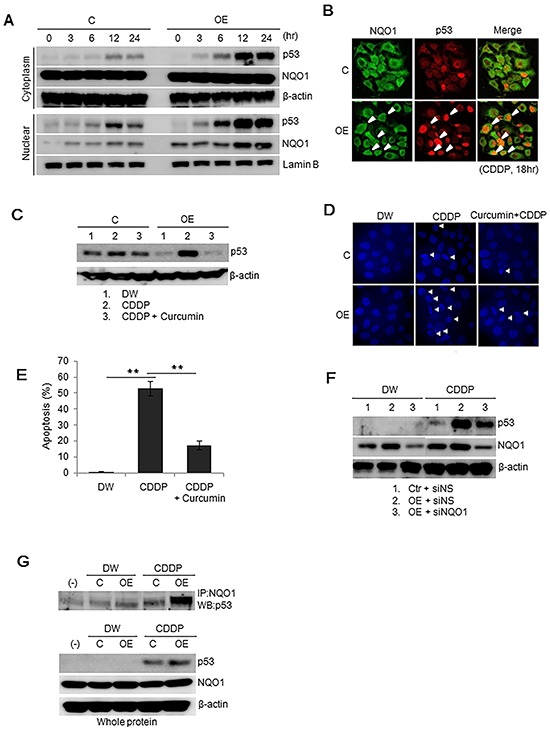
p53 in Ell3-OE treated with CDDP is stabilized via the NQO1-dependent pathway **A.** Nuclear translocation of p53 and NQO1 during CDDP treatment. The amounts of p53 and NQO1 protein in the cytosol and nucleus of control and Ell3-overexpressing (OE) cells were analyzed by western blotting at the indicated times after CDDP treatment. **B.** Co-localization of p53 and NQO1 in the nucleus after CDDP treatment. Control and Ell3-OE cells were treated with CDDP for 18 h and stained with antibodies to p53 (red) and NQO1 (green). Yellow color depicts co-localization of the two proteins. White arrows indicate the nuclear accumulation of NQO1 and p53. **C.** Inhibition of p53 accumulation by a chemical inhibitor of NQO1, curcumin. Accumulation of p53 protein in control and Ell3-OE cells pretreated with 50 μM curcumin was analyzed by western blotting after CDDP treatment for 6 h. **D.** Apoptotic cell death of control and Ell3-OE cells pretreated with curcumin was examined by DAPI staining 48 h after CDDP treatment. **E.** G0/G1 populations of control and Ell3-OE cells treated with curcumin and CDDP were stained with propidium iodide and analyzed by flow cytometry. **F.** Inhibition of p53 accumulation by siRNA-mediated suppression of NQO1 expression. Accumulation of p53 in control and Ell3-OE cells transfected with non-specific siRNA (siNS) or siRNA targeting Ell3 (siEll3) for 24 h was analyzed by western blotting after CDDP treatment for 6 h. **G.** Increased binding of NQO1 to p53 in Ell3-OE cells after CDDP treatment. Total protein of control and Ell3-OE cells treated with CDDP for 12 h, immunoprecipitated with anti-NQO1 antibodies, and then immunoblotted with anti-p53 antibodies. The error bars represent the SE from three independent experiments, each performed with triplicate samples. *P < 0.05, **P < 0.01, Student's *t*-test.

Figure 3C revealed that *MDM2* mRNA levels increased starting at 12 h after CDDP treatment in control cells. By contrast, p53 in Ell3-OE began to accumulate at 6 h after CDDP treatment, suggesting that another pathway stabilizing p53, independent of the ubiquitination pathway, operates in Ell3-OE cells. Recently, p53 was shown to be stabilized by NQO1 under stress conditions. p53 binding to NQO1 allows p53 to escape ubiquitin-independent 20S proteasomal degradation by transferring p53 to the nucleus [21]. Figure 4A shows that NQO1 gradually translocated to the nucleus after CDDP treatment and that higher amounts of NQO1 accumulated in the nucleus of Ell3-OE cells than in control cells. To confirm that NQO1 participates in the accumulation of p53, we examined the nuclear colocalization of NQO1 and p53 by fluorescence microscopy. As expected, a large proportion of Ell3-OE cells showed colocalization of p53 and NQO1 in the nucleus after CDDP treatment (Figure 4B).

Curcumin is an inhibitor of NQO1 that competes with NAD(P)H for binding to NQO1. It disrupts NQO1-p53 binding and thus induces ubiquitin-independent proteasomal p53 degradation [5, 6, 21, 22]. When Ell3-OE were pretreated with curcumin, CDDP-induced p53 accumulation was abolished and the level of p53 returned to basal levels (Figure 4C). Consistent with the decrease in the p53 protein level, curcumin pretreatment prevented CDDP-induced apoptotic cell death in Ell3-OE cells, as shown by their nuclear morphology, G0/G1 populations, and the results of the MTT assay (Figure 4D, 4E, Supplementary Figure S6). Inhibition of *NQO1* expression in Ell3-OE cells after the introduction of siNQO1 attenuated CDDP-induced p53 stabilization (Figure 4F). These results suggest that the accumulation of p53 in Ell3-OE cells in response to CDDP also depends on NQO1, which helps translocate p53 to the nucleus where it is protected from 20S proteasome degradation. Because NQO1 protects p53 by binding to p53, we analyzed whether this binding was stronger in Ell3-OE cells following CDDP treatment. As expected, immunoprecipitation analysis showed that the amount of p53 bound to NQO1 greatly increased following CDDP treatment (Figure 4G). These results indicate that Ell3 increases p53 stability by promoting NQO1 binding to p53 following CDDP treatment.

### Ell3 stabilizes p53 through IL20–ERK signaling following CDDP treatment

**Figure 5:**
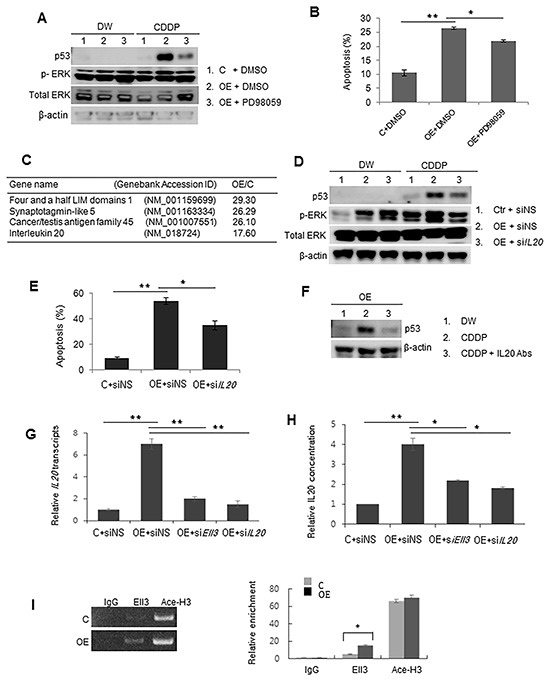
p53 in Ell3-OE is stabilized by ERK signaling activated by IL20 after CDDP treatment **A.** To confirm ERK phosphorylation, control (C) and Ell3-overexpressing (OE) MCF7 cells were pretreated with the ERK inhibitor PD980589 (50 μM) or DMSO vehicle for 24 h and p53 levels were analyzed by western blotting 16 h after CDDP treatment. **B.** The effect of PD98059 on apoptosis of control and Ell3-OE cells was quantified by annexin V staining and flow cytometry at 48 h after CDDP treatment. **C.** The five most up-regulated genes in ELL-OE cells, compared with control cells, determined by microarray analysis. **D.** The effect of si*IL20* on ERK phosphorylation and p53 stabilization in Ell3-OE cells after CDDP treatment. Non-specific siRNA (siNS) or siRNA targeting *IL20* (si*IL20*) was transfected into control and Ell3-OE cells for 24 h. Total p53 and phospho-ERK were analyzed by western blotting at 16 h after CDDP treatment. **E.** The effect of si*IL20* on the apoptosis of control and Ell3-OE cells following CDDP treatment for 48 h was quantitatively measured by flow cytometry. **F.** The effect of anti-IL20 antibodies added to the culture media of Ell3-OE on CDDP-induced p53 protein accumulation. To neutralize IL20, 4 μg IL20 antibody was added to the medium containing 6 × 10^4^ cells for 4 h, and the p53 protein level was analyzed by western blotting at 4 h after CDDP treatment with culture medium containing 1% FBS. **G.** The effect of si*Ell3* on the expression of *IL20* in Ell3-OE cells was analyzed by real-time PCR. Total RNA was analyzed 48 h after siRNA transfection. **H.** The effect of si*Ell3* and si*IL20* on the amount of secreted IL20 from Ell3-OE cells. After 24 h of siRNA transfection, IL20 in the culture media of control and Ell3-OE cells was measured using an IL20 immunoassay kit. **I.** Chromatin immunoprecipitation analysis of the transcription initiation region of the *IL20* promoter in control and Ell3-OE using antibodies against IgG (negative control), Ell3, and acetylated histone H3 (Ace-H3). Binding of Ell3 to the promoter of *IL20* was quantified by PCR and real-time PCR. The error bars represent the SE from three independent experiments, each performed with triplicate samples. *P < 0.05, **P < 0.01, Student's *t*-test.

Our results suggest that CDDP treatment of Ell3-OE cells activates a mechanism that triggers the rapid binding of NQO1 to p53. Previously, activation of ERK signaling was shown to be associated with increased resistance to 5-fluorouracil in Ell3-OE cells [17]. We therefore asked whether ERK signaling is also involved in the increased sensitivity of Ell3-OE cells to CDDP. Figure 5A shows that ERK signals in Ell3-OE cells were further activated following CDDP treatment and, when ERK signaling was chemically inhibited, CDDP-induced p53 accumulation was dramatically reduced. Consistent with the decrease in the p53 protein level, apoptotic cell death was also blocked when CDDP treatment was done in the presence of an ERK inhibitor (Figure 5B, Supplementary Figure S7A). This result indicates that ERK signaling mediates the stabilization of p53 in Ell3-OE cells after CDDP treatment.

We next asked what causes the increase in ERK signaling in Ell3-OE cells. To answer this question, we compared the gene expression profiles of Ell3-OE and control cells. Among the 143 genes whose expression was activated by more than 2-fold in Ell3-OE cells, *IL20* expression was the fifth highest (Figure 5C). Because Ell3-OE cells show increased colony formation and cell proliferation after ERK activation [17], we reasoned that IL20 might be the cause of the higher ERK signaling in Ell3-OE cells. To test this hypothesis, we suppressed *IL20* expression in Ell3-OE via siRNA and examined the ERK signal activation after CDDP treatment. Notably, introduction of si*IL20* into Ell3-OE cells decreased p53 protein accumulation and the phosphorylation of ERK1/2 following CDDP treatment (Figure 5D). Consistent with the reduced p53 accumulation, suppression of *IL20* expression by siRNA decreased apoptosis in Ell3-OE cells with CDDP treatment (Figure 5E, Supplementary Figure S7B). In addition, depletion of IL20 in the culture media using an anti-IL20 monoclonal antibody substantially decreased CDDP-induced accumulation of p53 and the apoptosis of Ell3-OE cells (Figure 5F).

When *Ell3* expression was suppressed by si*Ell3* in Ell3-OE cells, *IL20* expression was decreased (Figure 5G). Consistently, the amount of secreted IL20 was also decreased by the suppression of *Ell3* expression (Figure 5H). These results suggest that *IL20* expression is dependent on the expression level of *Ell3*. To examine whether Ell3 directly increased *IL20* transcription, we conducted ChIP analysis of the *IL20* promoter using the Ell3 antibody. The transcription initiation site of *IL20* was enriched by more than 2-fold in Ell3-OE cells compared with control cells (Figure 5I). Taken together, these results suggest that the chemosensitive response of Ell3-OE cells is mainly due Ell3 functioning as a transcriptional activator to increase the transcriptional efficiency of *IL20*, resulting in higher Il20 levels and the activation of IL20-induced ERK1/2 signaling.

## DISCUSSION

**Figure 6:**
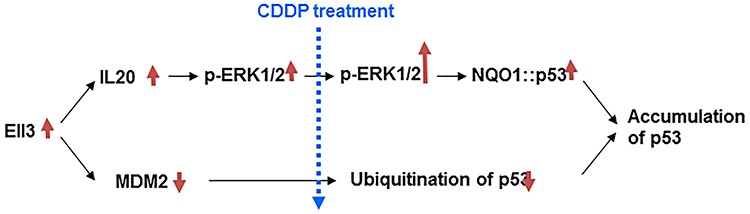
Model for Ell3 activity stabilizing p53 protein following CDDP treatment in breast cancer cells Ell3 expression leads to the suppression of MDM2 expression and activation of the IL20 mediated ERK1/2 signaling pathway. Due to the low level of MDM2, p53 is not ubiquitinated following CDDP treatment. Additional ERK1/2 is phosphorylated following CDDP treatment, resulting in increased binding affinity of NQO1 to p53. The synergistic effect of suppressing the MDM2 pathway and enhancing the IL20 pathway causes rapid stabilization of p53 in Ell3-overexpressing breast cancer cells following CDDP treatment.

This study demonstrates that Ell3 induces a chemosensitive response in breast cancer cell line MCF7, by suppressing the MDM2-mediated pathway of p53 ubiquitination and increasing the binding of p53 to NQO1 after CDDP treatment (Figure 6).

In the xenograft mouse model, Ell3-OE cells formed fewer tumors that were much smaller than the tumors formed by control MCF7 cells (Supplementary Figure S8, left). p53 accumulation in Ell3-OE tumors (Supplementary Figure S8, right) suggests that these cells underwent apoptosis. Because IL20 is a proinflammatory cytokine involved in various inflammatory diseases and ischemic stroke [9, 13], it will be interesting to assess whether Ell3-OE recruits immune cells that cause p53-induced apoptosis.

Previous work [8, 23] and our own studies [17] reveal that the ERK signaling pathway in Ell3-OE cells is the main gateway for p53 stabilization following CDDP treatment. Chemical inhibition of ERK signaling suppressed CDDP-mediated apoptosis and p53 accumulation in Ell3-OE cells. ERK signaling activated by the higher level of IL20 in Ell3-OE cells was further stimulated by CDDP treatment. Because increased ERK1/2 signaling in Ell3-OE cells did not have any effect on p53 stabilization during normal culture conditions, we speculate that the abnormally high levels of ERK signaling in Ell3-OE cells preconditioned them to rapidly respond to CDDP. However, we cannot rule out the possibility that other factors, in addition to ERK signaling, are synergistically involved in the hypersensitive CDDP response of Ell3-OE cells. Catalase and glutathione S-transferase, enzymes, which balance the redox status of a cell, are transcriptionally suppressed in Ell3-OE cells (data not shown). Another factor potentially involved in the CDDP response could be reactive oxygen species (ROS), which are present at high levels in Ell3-OE cells (Supplementary Figure S9A). ROS stabilize p53 under hypoxic conditions [24], and in our experiment, pretreatment of Ell3-OE with a ROS scavenger, sodium pyruvate decreased p53 accumulation in CDDP-treated Ell3-OE cells (Supplementary Figure S9B). This result indicates that the high level of ROS in Ell3-OE cells also contributes to the hypersensitive response of Ell3-OE cells to CDDP.

p53 protein stability is mainly controlled by dynamic and reversible ubiquitination, which sensitizes p53 protein to 26S proteosomal degradation. Our results suggest that Ell3 stabilizes p53 via its effects on both ubiquitin-dependent and -independent mechanisms. The results in Figure 3 show that ubiquitin-dependent degradation of p53 is impaired in Ell3-OE cells. This could be explained by increased MDM2 protein degradation in Ell3-OE cells. Indeed, p53 bound to the *MDM2* promoter and activated *MDM2* transcription upon CDDP treatment; however, MDM2 did not accumulate in Ell3-OE cells. The simplest explanation for these contradictory results is that proteosomal degradation of MDM2 is activated in Ell3-OE cells, which would prevent the accumulation of MDM2 despite that the increase in its transcription.

In addition to the ubiquitin-dependent degradation pathway, p53 protein is also regulated by a constitutive ubiquitin-independent mechanism of degradation by the 20S proteasome. We showed that the binding affinity of p53 to NQO1 was enhanced by CDDP treatment of Ell3-OE cells. In addition, treatment with curcumin, which dissociates the NQO1–p53 complex, significantly decreased p53 accumulation and the apoptotic cell population in CDDP-treated Ell3-OE cells. These results suggest that the high levels of p53 accumulation following CDDP treatment in Ell3-OE cells are the result of an additive effect or a synergism between a decrease in ubiquitin-dependent degradation pathway activity and an increase in NQO1-dependent protection of p53 against degradation. Therefore, it is not surprising that the amount of p53 accumulation in the nucleus of CDDP-treated Ell3-OE was much higher than the amount of NQO1. NQO2 is also known to contribute to stabilization of p53 in the nucleus. However, NQO2 did not translocate to the nucleus in CDDP-treated Ell3-OE cells (data not shown), suggesting that the activities of NQO1 and NQO2 in stabilizing p53 are regulated by independent pathways.

Previously, we reported that ERK1/2 signaling in Ell3-OE cells mediates 5-fluorouracil resistance [17]. This result is in contrast to the sensitive response of Ell3-OE cells to CDDP, where ERK1/2 signaling promoted p53 stability upon CDDP treatment. The reasons for this discrepancy are unknown, but may be due to the opposite patterns of MDM2 accumulation and phospho-p53 (S15) accumulation in 5-fluorouracil- and CDDP-treated cells (Supplementary Figure S10). Elucidating how Ell3 induces contradicting responses in cancer cells to different drugs will extend our understanding of the regulatory mechanism of p53 stability.

In conclusion, we have demonstrated that the transcription elongation factor Ell3 induces chemosensitization of MCF7 cells to the chemotherapeutic agent CDDP by stabilizing p53. Our data suggest that p53 stabilization following treatment with an antitumor drug is a highly regulated process. The balance between degradation and stabilization is not simply determined by the ubiquitination status of p53, but rather is regulated by cytokine expression and signaling pathways that manage the ubiquitin-dependent and ubiquitin-independent degradation of p53. This knowledge will provide a basis for the development of rational therapeutic applications designed to induce chemosensitization of breast cancer cells.

## MATERIALS AND METHODS

### Cell culture and establishment of Ell3-overexpressing breast cancer cell lines

The MCF7 cell line was purchased from American Type Culture Collection (ATCC, Rockville, USA). Cells were cultured in DMEM containing 10% fetal bovine serum and 1% penicillin/streptomycin. Ell3-overexpressing breast cancer cell lines were established by chromosomal integration of an Ell3 expression plasmid, which was constructed by cloning the PCR-amplified Ell3 cDNA into a pcDNA3.1 vector (Invitrogen, Carlsbad, USA). Three Ell3-overexpressing cell lines were generated, and all experiments were done using each cell line to confirm the results.

### Apoptosis assay

An apoptosis detection kit providing fluorescein isothiocyanate (FITC)-conjugated annexin V and propidium iodide was used according to the manufacturer's protocol (LS-02–100; BioBud, Korea). Samples were analyzed by flow cytometry using FL1 (FITC) and FL2 (PI) laser lines.

### Western blotting

Cells were washed with phosphate-buffered saline (PBS) and lysed with tissue lysis buffer (20 mM Tris-base, pH 7.4, 137 mM NaCl, 2 mM EDTA, 1% Triton X-100, 25 mM β-glycerophosphate, 2 mM sodium pyrophosphate, 10% glycerol, 1 mM sodium orthovanadate, 1 mM benzamidine, and 1 mM phenylmethysulfonyl fluoride). Total cell extracts were resolved by sodium dodecyl sulfate polyacrylamide gel electrophoresis (SDS–PAGE), transferred to Immobilon-P membranes (Millipore, Bedford, USA), and blotted with antibodies against Ell3 (ab67415, Abcam, Cambridge, USA), p53 (#2524, Cell Signaling, Denver, USA), Caspase-9 (#9665, Cell Signaling), NQO1 (sc-16464, Santa Cruz Biotechnology, Dallas, USA), phospho-ERK (#4370, Cell Signaling), total ERK (sc-145, Santa Cruz Biotechnology), lamin B (sc-6216, Santa Cruz Biotechnology), ɤH2AX (ab22551, Abcam), MDM2 (sc-965, Santa Cruz Biotechnology) and β-actin (sc-47778, Santa Cruz Biotechnology). Immunoreactivity was detected by enhanced chemiluminescence (ECL; Bio-Rad, CA, USA).

### Immunoprecipitation

Cells were washed with PBS and harvested in lysis buffer. Cell lysates were cleared by centrifugation at 12,000 rpm for 15 min at 4°C. For immunoprecipitations, cell lysates were incubated with 4 μg of antibody for 16 h at 4°C followed by incubation with 30 μl of protein G beads for 1 h at 4°C. Beads were washed once in lysis buffer. Samples were boiled in SDS–PAGE sample buffer and analyzed by western blotting.

### GST pull-down assay

The plasmid expressing GST-p53 fusion protein was a generous gift from Dr. K.H. Baek (CHA University, Korea). HEK 293 cells were transfected with an Ell3 expression plasmid using Lipofectamine 2000 (Invitrogen) as described by the manufacturer. Cells were lysed in a Triton X-100 lysis buffer and incubated with GST–p53 beads overnight at 4°C. The beads were washed and centrifuged, and the bound proteins were eluted with 10 mM glutathione. The eluates were subjected to western blotting.

### Immunofluorescence staining

Cells were cultured on cover slips, washed twice with PBS, and fixed with 4% paraformaldehyde for 15 min. The cover slips were washed three times with PBS and permeabilized with 0.1% Tween-20 in PBS for 20 min followed by blocking for 30 min using blocking buffer (5% bovine serum albumin in PBS). After an overnight incubation with the primary antibodies at 4°C, the cover slips were washed three times with PBS and incubated with fluorescent secondary antibodies for 1 h in the dark at room temperature. The cover slips were then washed three times in PBS and mounted with Vectashield Mounting Medium with DAPI (H-1200, Vector Laboratories, Burlingame, USA). Images were acquired at room temperature using a confocal microscope (LSM 510 META; Carl Zeiss).

### mRNA expression

RNA was isolated using TRI-reagent (Sigma-Aldrich, St. Louis, USA). Total RNA (1 μg) was reverse-transcribed using the 1^st^ Strand cDNA Synthesis system (LeGene, San Diego, USA) according to the manufacturer's protocol. Real-time PCR was performed in triplicate using the primers listed in Supplementary Table S1, with the TOPreal qPCR 2X PreMIX (Enzynomics, Korea) and the CFX96 Real-time System (Bio-Rad Laboratories, Richmond, USA). Levels were normalized to that of glyceraldehyde 3-phosphate dehydrogenase (GAPDH).

### Cell cycle analysis

Cells were prepared as a single-cell suspension of 1 × 10^6^ cells/ml in PBS. After fixing with cold 70% ethanol for 2 h, cells were washed with cold PBS and then stained with propidium iodide at a final concentration of 50 μg/ml in the presence of 20 μg/ml RNase and 10% Triton X-100 in PBS for 1 h at 37°C. Treated cells were washed with PBS and then evaluated by flow cytometry.

### Transfection with siRNA

MCF7 cells were transfected with siRNA using SiGENOME (*Ell3*, M-014601-01-0005; *TP53*, L-003329-00-0005; *IL20*, L-007968-00-0005; *NQO1*, L-005133-00-0005), which was provided by Dharmacon (distributed by ThermoScientific/AbGen, Epsom, UK). Cells were transfected with either siRNA or non-specific siRNA using Lipofectamine 2000 (Invitrogen) in OPTI-MEM (Invitrogen) according to the manufacturer's instructions.

### Quantification of IL20 in culture media

A human IL20 immunoassay kit was used (DL200, R&D Systems). Optical density of each well was determined using a microplate reader set to 450 nm.

### Microarray gene expression analysis

Total RNA was extracted using TRIzol (Invitrogen) and used to prepare biotinylated cRNA using the Illumina TotalPrep RNA Amplification Kit (Ambion, Austin, USA). Following fragmentation, cRNA was hybridized to Illumina HumanHT-12 v4 Expression BeadChips according to the manufacturer's instructions (BD-103-0204, Illumina, USA). Arrays were scanned with an Illumina Bead Array Reader Confocal Scanner. Array data processing and analysis were performed using Illumina Bead Studio v3.1.3 (Gene Expression Module v3.3.8).

### Chromatin immunoprecipitation (ChIP) assay

The ChIP assay was performed to analyze the binding activity of p53 protein to the *MDM2* promoter and the binding of Ell3 to the *IL20* promoter. 1% formaldehyde was added to the cell culture media for 10 min at 37°C. Cells were washed three times with cold PBS and then resuspended in lysis buffer (1% SDS, 10 mM EDTA, 50 mM Tris-HCl, pH 8.1) with 1 mM phenylmethylsulfonyl fluoride (PMSF). After a brief sonication, lysates were cleared by centrifugation and diluted 5-fold with dilution buffer (0.01% SDS, 1% Triton X-100, 1.2 mM EDTA, 16.7 mM Tris-HCl, pH 8.1, 167 mM NaCl) containing PMSF. Cell lysates were incubated with anti-p53 antibody or anti-Ell3 antibody overnight at 4°C. Immune complexes were precipitated with Protein A/G Plus Agarose. Precipitants were sequentially washed with low-salt wash buffer (0.1% SDS, 1% Triton X-100, 2 mM EDTA, 20 mM Tris-HCl, pH 8.1, 150 mM NaCl), high-salt wash buffer (0.1% SDS, 1% Triton X-100, 2 mM EDTA, 20 mM Tris-HCl, pH 8.1, 500 mM NaCl) and LiCl wash buffer (0.25 M LiCl, 1% NP-40, 1% deoxycholate, 1 mM EDTA, 10 mM Tris-HCl, pH 8.1). After the final wash, elution buffer (1% SDS, 0.1 M NaHCO_3_) was added and incubated at room temperature for 15 min with rotation. Then, the formaldehyde crosslinking was reversed by adding 0.3 M NaCl and heating at 65°C for 4 h. Proteinase K was added at 45°C for 1 h. DNA was recovered by phenol–chloroform extraction and ethanol precipitation. Pellets were resuspended in TE buffer and subjected to PCR using primers for the promoter of *MDM2* or *IL20*. The PCR product was separated by agarose gel electrophoresis.

### *In vivo* tumor growth assay

Female 6-week-old BALB/c nude mice were implanted with estradiol (E2) pellets (1.7 mg per pellet, 60-day release) in the intrascapular region. One day later, Ell3-OE or control MCF7 cells were surgically implanted into a mouse's mammary fat pad (5.0 × 10^6^ cells per injection site). Eight weeks later, tumors were dissected from the mice and analyzed for size and p53 expression. Animal experiments were approved by the Committee of Laboratory Animal Care of the CHA University.

### Statistical analysis

Each experiment was performed at least three times. Statistical significance between two groups was determined using Student's *t*-test, and a *P* value < 0.05 was considered significant. All statistical analyses were performed using the SAS statistical package, v.9.13 (SAS, Cary, USA).

## SUPPLEMENTARY FIGURES AND TABLE


