# Supplementary material for: Ell3 stabilizes p53 following CDDP treatment via its effects on ubiquitin-dependent and -independent proteasomal degradation pathways in breast cancer cells
Source: Oncotarget. 2015 Oct 19;6(42):44523–37. doi: 10.18632/oncotarget.5972 (PMC4792573; doi:10.18632/oncotarget.5972)
Supplement: Supplementary file 1 [file oncotarget-06-44523-s001.pdf]

SUPPLEMENTARY FIGURES AND TABLE

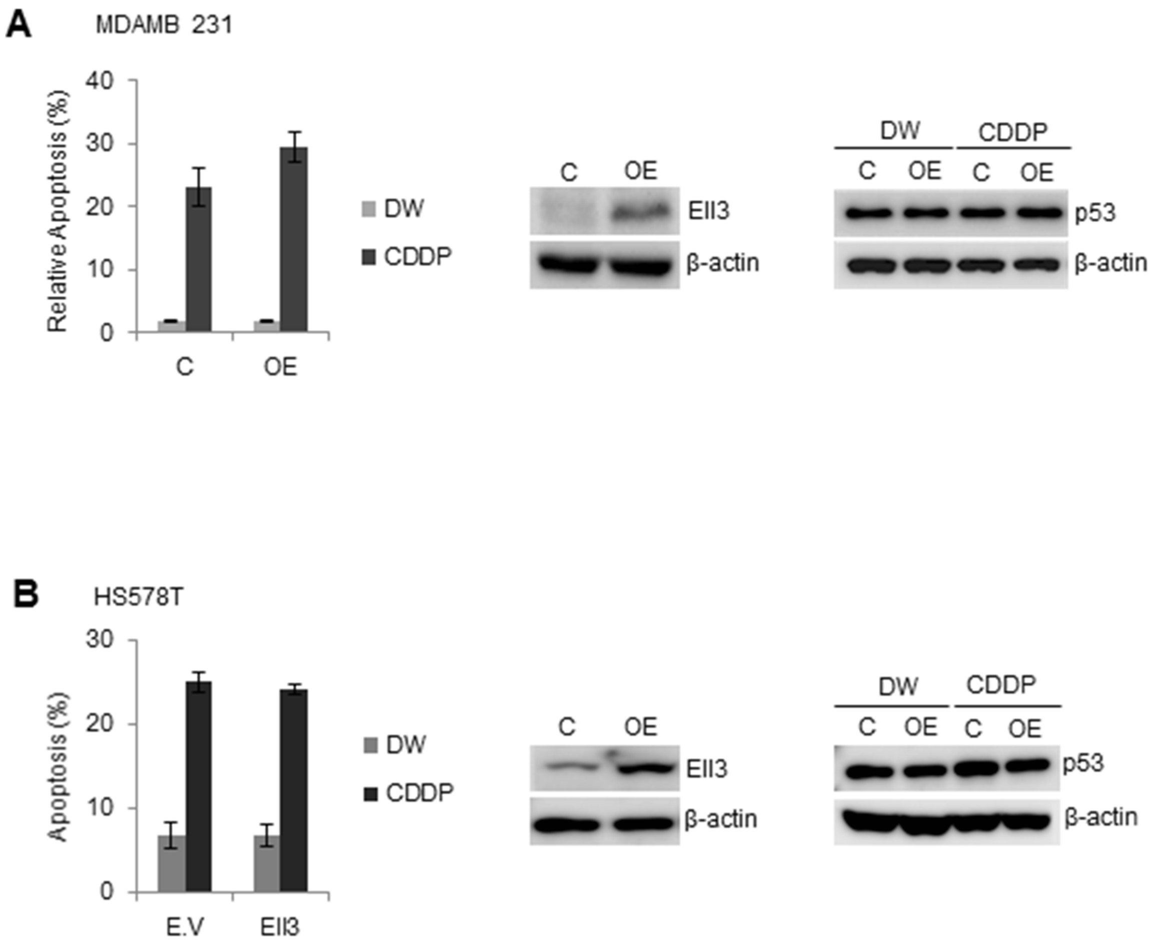

Supplementary Figure S1: The response of MDA-MB-231 and Hs578T cells overexpressing Ell3 to CDDP. MDA-MB-231 and Hs578T cells were engineered to overexpress Ell3 by the transduction of an Ell3-expressing retrovirus. The apoptotic response was analyzed by annexin V staining and flow cytometry at 48 h after CDDP treatment. Ell3 expression levels and p53 accumulation were analyzed by western blotting with samples harvested 48 h after CDDP treatment.

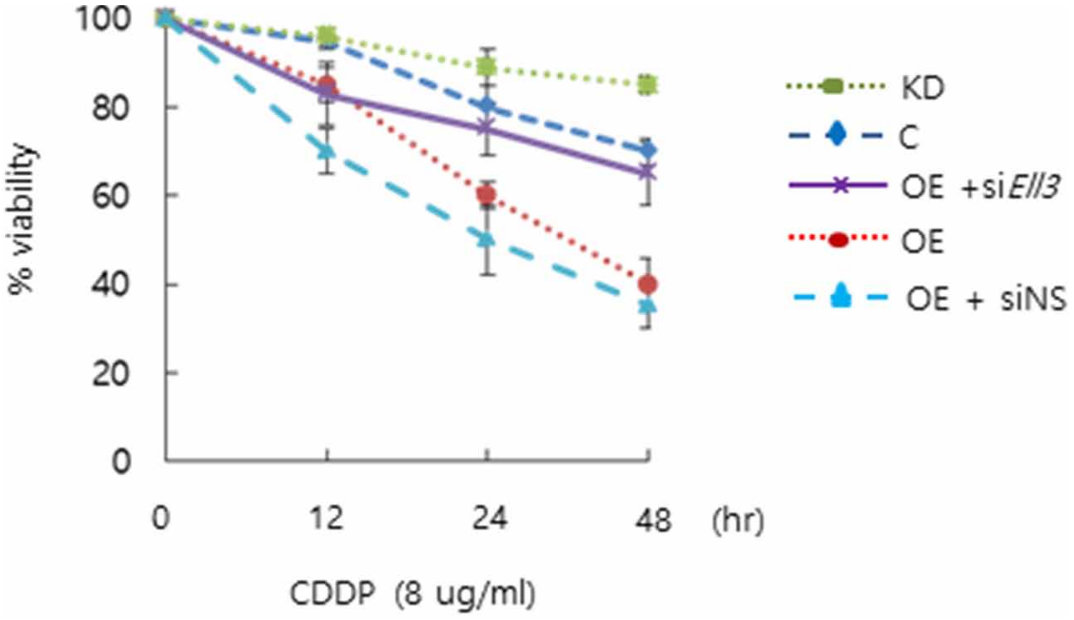

Supplementary Figure S2: MTT assay of Ell3 OE, KD, OE + siRNA.

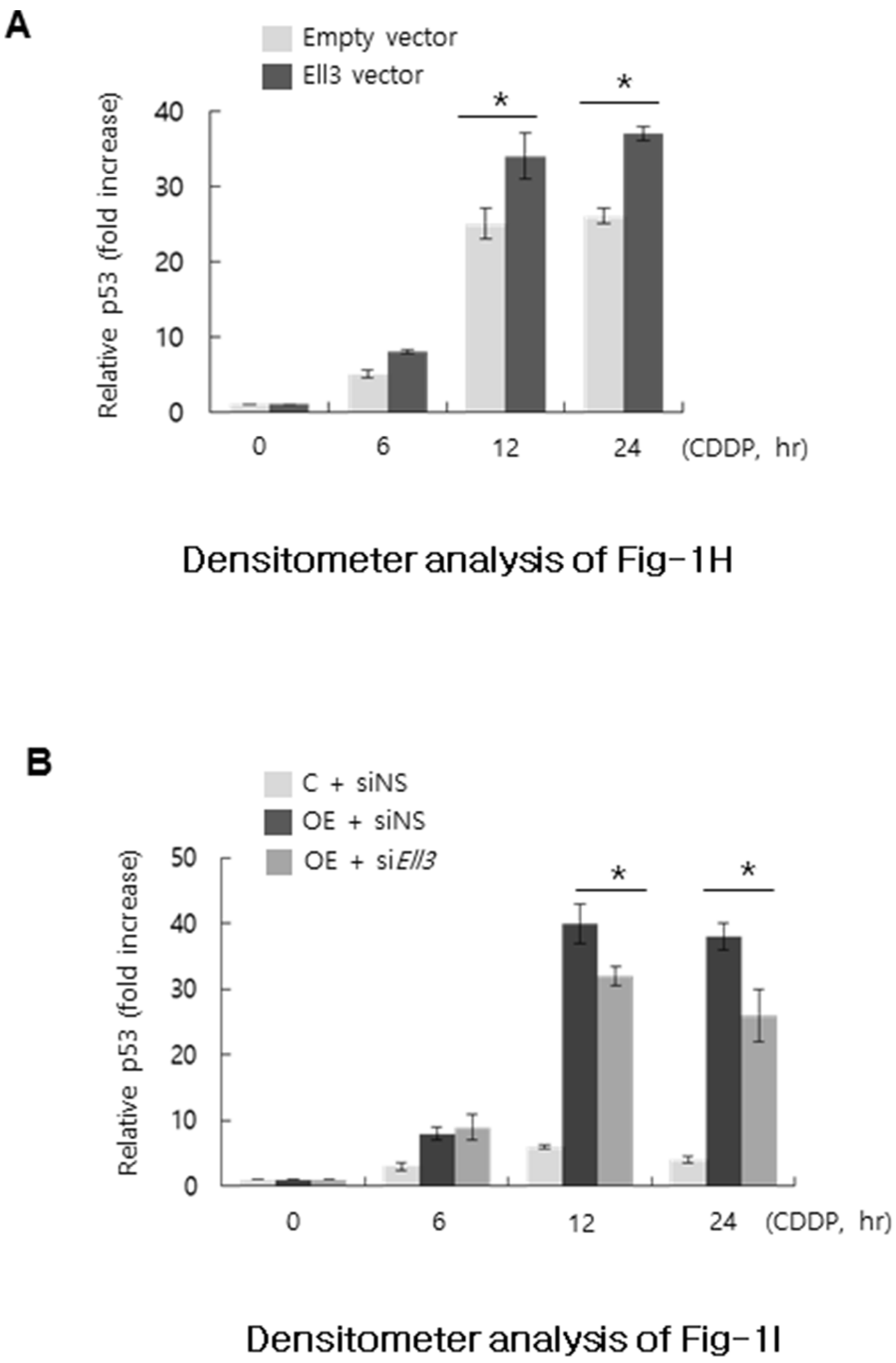

Supplementary Figure S3: Densitometric analysis of the data in Figure 1H and 1I.

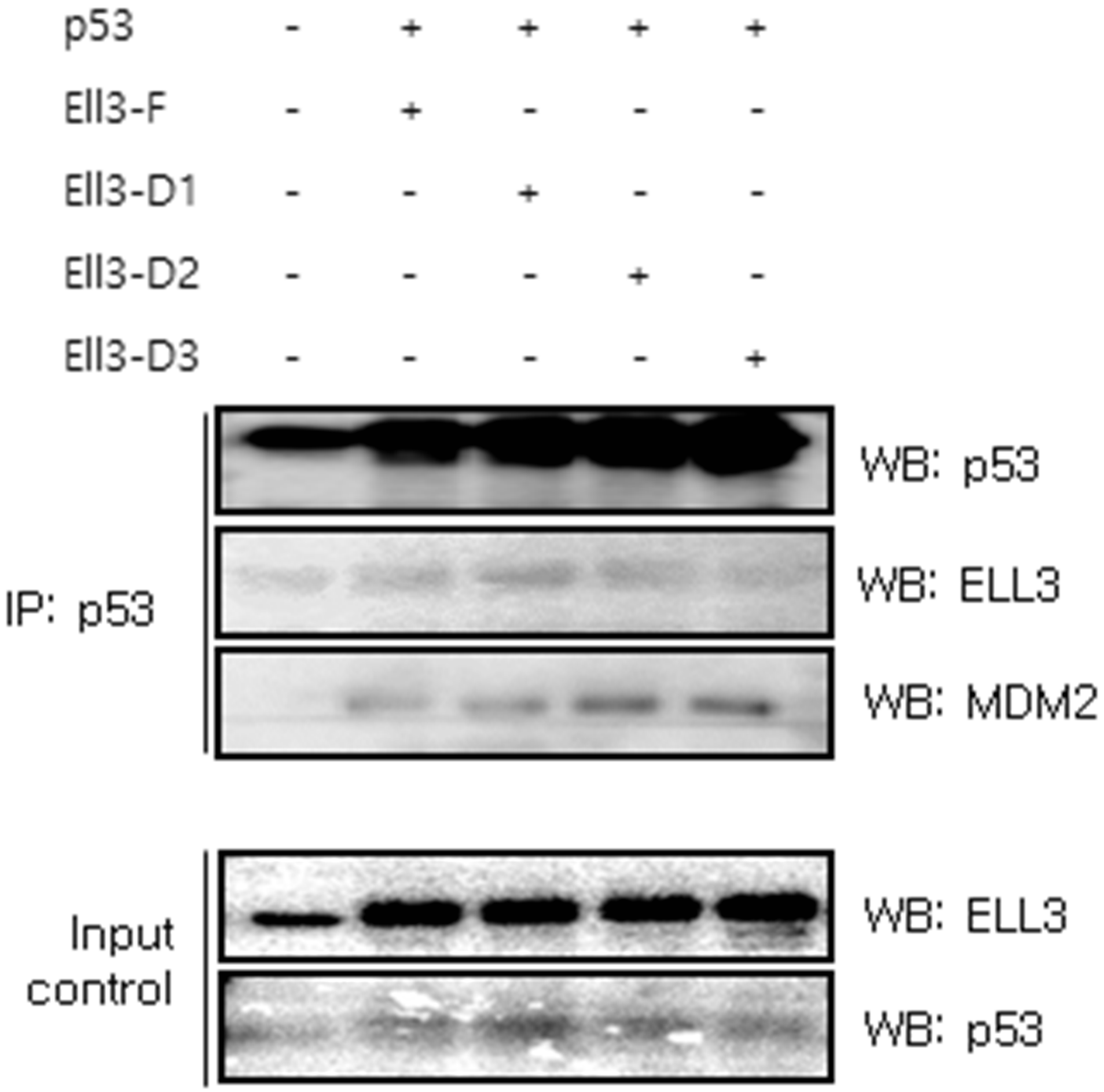

Supplementary Figure S4: Co-immunoprecipitation analysis of p53 and deleted mutants of Ell3. Cell lysates collected from HEK293 cells transfected with plasmids expressing truncated Ell3 and p53 were immunoprecipitated with an anti-p53 antibody and immunoblotted with anti-Ell3 antibody. *Ell3-F*, full-length Ell3 (residues 1–397); *Ell3-D1*, Ell3 deletion mutant 1 (residues 100–397); *Ell3-D2*, Ell3 deletion mutant 2 (residues 200–397); *Ell3-D3*, Ell3 deletion mutant 3 (residues 300–397).

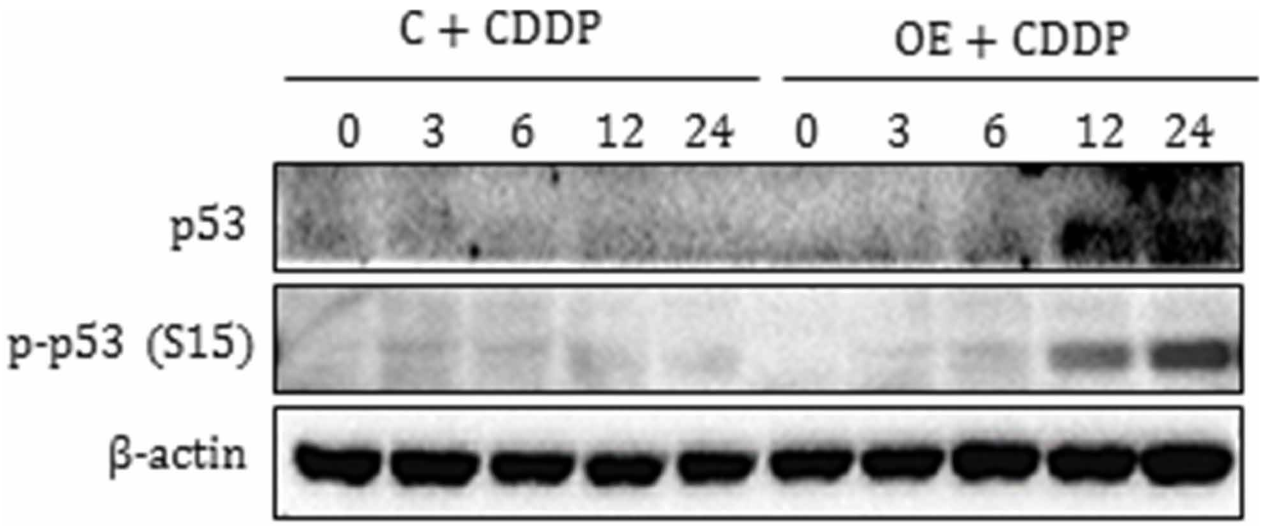

Supplementary Figure S5: Accumulation of phospho-p53 (S15) in control or Ell3-OE cells analyzed by western blotting at the indicated times after CDDP treatment.

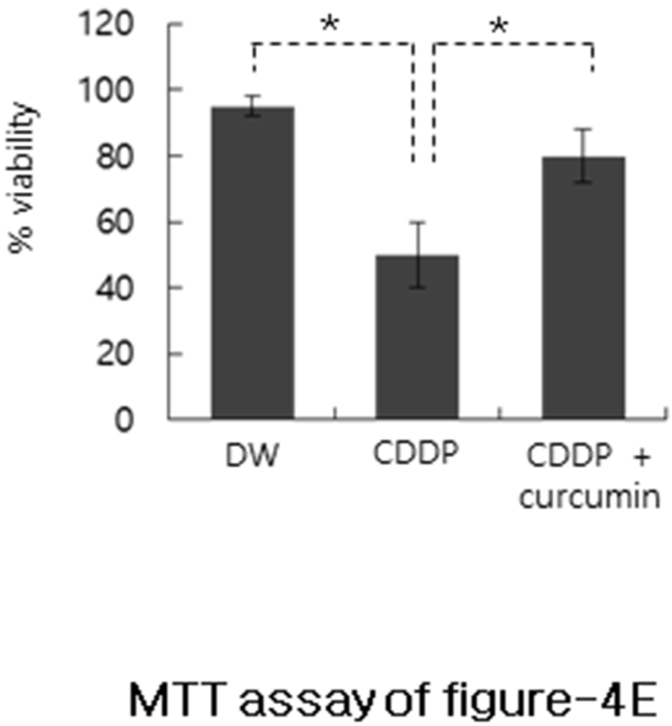

Supplementary Figure S6: MTT assay of samples shown in Figure 4E.

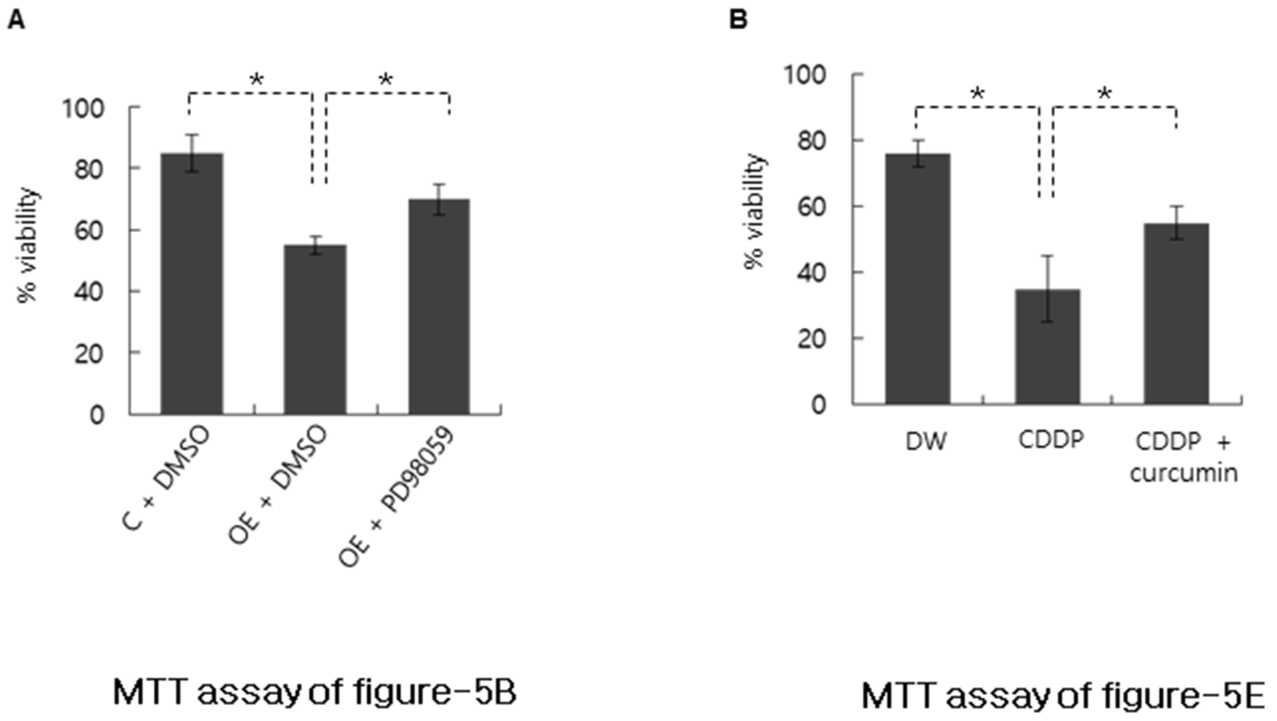

Supplementary Figure S7: MTT assay of samples shown in Figure 5B A. and Figure 5E B.

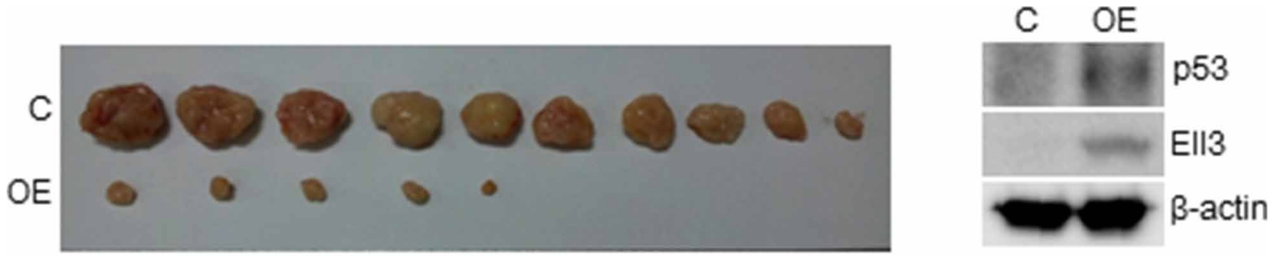

Supplementary Figure S8: Effect of Ell3 overexpression on teratoma formation *in vivo*. Ell3-OE and control MCF7 cells were injected into BALB/c nude mice to assay for teratoma formation. Eight weeks after injection, Ell3-OE cells formed larger teratomas than control cells. p53 protein was detected in the teratoma of Ell3-OE cells.

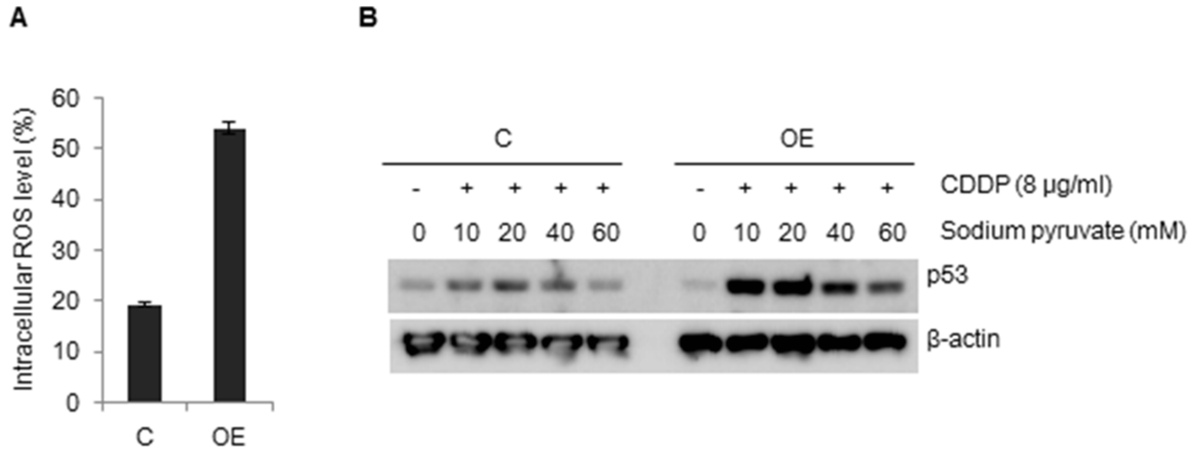

Supplementary Figure S9: Correlation of ROS and p53 protein amount in Ell3-OE. A. By DCF-DA staining and flow cytometry, reactive oxygen species (ROS) were found to be present at higher levels in Ell3-overexpressing (OE) cells than in control cells (C). **B.** The effect of indicated concentrations of sodium pyruvate on CDDP-mediated accumulation of p53 in Ell3-OE analyzed by western blotting. CDDP treatment was for 18 h.

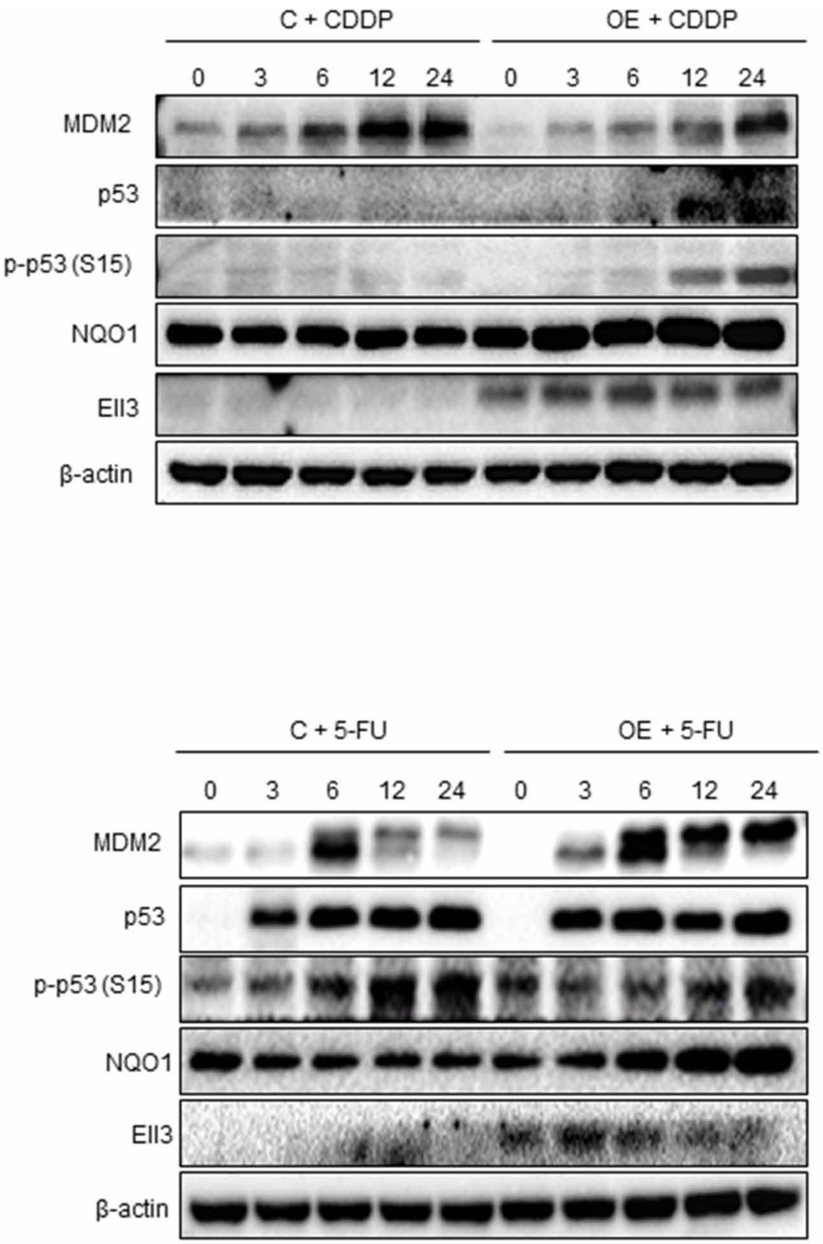

Supplementary Figure S10: Western blotting of MDM2, p53, phospho-p53 (S15), and NQO1 in control and Ell3-OE cells at the indicated times after CDDP or 5-fluorouracil (5-FU) treatment.

Supplementary Table S1: PCR primers

Gene	Primer sequence (5′ to 3′)
GAPDH	F-ACC ACA GTC CAT GCC ATC AC
	R-TCC ACC ACC CTG TTG CTG TA
Ell3	F-CTA CAA GGC CTG ACC AAT CAG G
	R-CTG GAG TTC CTC GCC GAA CTC
NOXA	F-GAG ATG CCT GGG AAG AAG G
	R-TCC TGA GCA GAA GAG TTT GGA
GADD45A	F-ACG AGG ACG ACG ACA GAG AT
	R-GCA GGA TCC TTC CAT TGA GA
p21	F-GAC ACC ACT GGA GGG TGA CT
	R-CAG GTC CAC ATG GTC TTC CT
Bax	F-CAG TTG AAG TTG CCG TCA GA
	R-ACG AGG ACG ACG ACA GAG AT
FAS	F-CAA GGG ATT GGA ATT GAG GA
	R-TGG AAG AAA AAT GGG CTT TG
PIG3	F-TAG CCG TGC ACT TTG ACA AG
	R-ATG CCT CAA GTC CCA AAA TG
MDM2	F-GGT GGG AGT GAT CAA AAG GA
	R-ACA CAG AGC CAG GCT TTC AT
WWP1	F-GCT TAT GAA CGC GGC TTT AG
	R-AAG CGC CTC CTC AAG TCA TA
IL20	F-CGC CAA TTC CTT TCT TAC CA
	R-CTA GTT CCC CCA AAG CCT TC
IL20 (ChIP)	F-CTG TGG GTG GCC ATA AAA TC
	R-AAA ACG CAG CA GAGA GAA GG
MDM2 (ChIP)	F-TGG GCA GGT TGA CTC AGC TTT TCC TC
	R- TTC CGA AGC TGG AAT CTG TGA CCT GC
